# Two-dimensional Ti_3_C_2_T_x_ MXene promotes electrophysiological maturation of neural circuits

**DOI:** 10.1186/s12951-022-01590-8

**Published:** 2022-08-31

**Authors:** Yige Li, Yangnan Hu, Hao Wei, Wei Cao, Yanru Qi, Shan Zhou, Panpan Zhang, Huawei Li, Geng-Lin Li, Renjie Chai

**Affiliations:** 1 State Key Laboratory of Bioelectronics, Department of Otolaryngology Head and Neck Surgery, Zhongda Hospital, School of Life Sciences and Technology, Advanced Institute for Life and Health, Jiangsu Province High-Tech Key Laboratory for Bio-Medical Research, Southeast University, Nanjing, 210096 China; 2 Department of Otolaryngology Head and Neck Surgery, Affiliated Drum Tower Hospital of Nanjing University Medical School, Nanjing, 210008 China; 3 Department of Otolaryngology Head and Neck Surgery, Sichuan Provincial People’s Hospital, University of Electronic Science and Technology of China, Chengdu, 610072 China; 4 Co-Innovation Center of Neuroregeneration, Nantong University, Nantong, 226001 China; 5 Institute for Stem Cell and Regeneration, Chinese Academy of Science, Beijing, 100086 China; 6 Beijing Key Laboratory of Neural Regeneration and Repair, Capital Medical University, Beijing, 100069 China; 7 Department of Otorhinolaryngology, Head and Neck Surgery, The Second Hospital of Anhui Medical University, Hefei, 230069 China; 8 Department of Otorhinolaryngology and ENT Institute, Eye and ENT Hospital, Fudan University, Shanghai, 200031 China; 9 State Key Laboratory of Medical Neurobiology, and MOE Frontiers Center for Brain Science, Fudan University, Shanghai, 200031 China; 10 Institutes of Biomedical Sciences, Fudan University, Shanghai, 200032 China; 11 NHC Key Laboratory of Hearing Medicine Fudan University, Shanghai, 200031 China; 12 Institutes of Brain Science and Collaborative Innovation Center for Brain Science, Fudan University, Shanghai, 200032 China

**Keywords:** Neural stem cells, Neuronal development and maturation, Ti_3_C_2_T_x_ MXene, Patch-clamp recording, Neural spiking, Synaptic transmission

## Abstract

**Background:**

The ideal neural interface or scaffold for stem cell therapy shall have good biocompatibility promoting survival, maturation and integration of neural stem cells (NSCs) in targeted brain regions. The unique electrical, hydrophilic and surface-modifiable properties of Ti_3_C_2_T_x_ MXene make it an attractive substrate, but little is known about how it interacts with NSCs during development and maturation.

**Results:**

In this study, we cultured NSCs on Ti_3_C_2_T_x_ MXene and examined its effects on morphological and electrophysiological properties of NSC-derived neurons. With a combination of immunostaining and patch-clamp recording, we found that Ti_3_C_2_T_x_ MXene promotes NSCs differentiation and neurite growth, increases voltage-gated current of Ca^2+^ but not Na^+^ or K^+^ in matured neurons, boosts their spiking without changing their passive membrane properties, and enhances synaptic transmission between them.

**Conclusions:**

These results expand our understanding of interaction between Ti_3_C_2_T_x_ MXene and NSCs and provide a critical line of evidence for using Ti_3_C_2_T_x_ MXene in neural interface or scaffold in stem cell therapy.

**Supplementary Information:**

The online version contains supplementary material available at 10.1186/s12951-022-01590-8.

## Background

With latest advances in biological sciences, transplantation of neural stem cells (NSCs) shows great potential for treating neurodegenerative diseases. In order for this strategy to be successful, transplanted NSCs must replace ailing and dead neurons and form neural connections with remaining healthy neurons, to a significant extent so that at least part of original neural functions can be restored [1, 2]. Therefore, it is critically important to promote NSC development and maturation in targeted brain region [3]. However, low survival rate, unwanted cell differentiation and lack of functional integration are among the biggest obstacles for clinical application of NSCs in therapy [4, 5].

The brain consists of a complex network of neurons located in the extracellular matrix (ECM) with heterogenous components and three-dimensional structures [6]. Under physiological conditions, ECM is composed of proteins and polysaccharides, secreted and assembled by cells, providing channels for them to send and receive chemical, electrical and mechanical signals [7, 8]. In cell transplantation, extracellular matrix proteins are shown to possess biological properties in guiding cell behavior and/ or tissue regeneration but often lack sufficient mechanical properties. However, these properties are required for biomaterials and physically guide cell fate and function [9] through elastic modulus, electrical conductivity, topology, spatial dimension, and dynamic degradability, such as cell adhesion [10], migration [11], proliferation [12], differentiation [13] and stem cell maintenance [14]. It has been shown that the composition, physical properties, surface and structural features of biomaterials can modulate cell behavior and guide the process of tissue regeneration, mainly through the direct interaction between cells and biomaterials [15, 16].

Ion channels are essential for regulating the physiological function of cells and maintaining the stability of the intracellular environment. Because of their unique molecular structure and contact sites, ion channels have become potential targets for extracellular stimuli, including synthetic drugs and biomaterials [17]. In recent years, many studies have reported that biomaterials can interact with ion channels directly and / or indirectly, change ion homeostasis, ion channel currents, channel dynamics, ion channel-related RNA and protein expression levels, and trigger a variety of intracellular signal transduction pathways [17–19].

Among the biomaterials tested so far, graphene [20–23], carbon nanotubes [24, 25] and their derivatives have been widely used to create microenvironment for neural stem cells, and have shown the potential to promote nerve regeneration and the ability to regulate ion currents [26–30]. In addition, their excellent thermal, electrical and mechanical properties make them show great prospects in terms of high-resolution and stable neural interfaces [31]. However, the hydrophobicity of these carbon-based materials limits their surface functionalization and further applications [32].

In contrast, Ti_3_C_2_T_x_ MXene, a new kind of two-dimensional (2D) crystal nanomaterial in a general formula of M_n+1_X_n_T_x_, represents a new and more promising direction. Due to the presence of transition metals (M in the formula, such as Ti and Zr) and surface groups (T in the formula, such as O^2−^ and OH^−^), Ti_3_C_2_T_x_ MXene is not only conductive but also hydrophilic [33, 34], affording rich anchoring sites and modifiability and making it attractive for many applications, including nanomedicine [35–40], biosensor [41–45], antimicrobial therapy [46, 47], self-cleaning [48, 49], biological imaging [50, 51] and therapeutic diagnostics [52, 53].

With the increasing research on MXenes, these materials have been successfully applied in the field of biomedicine [54]. 2D structures of MXenes with abundant surface anchoring sites have potential to be used as drug or protein carriers [55]. MXenes is considered as a high potential antimicrobial agent for inhibiting the growth of bacteria and fungi because of its large specific surface, operable surface functionalization and the potential to load different antibacterial functional groups [47, 56]. MXenes absorbs non-toxic electromagnetic radiation in the near infrared spectrum and is used in photothermal therapy and photoacoustic imaging of cancer [57, 58]. Due to the interaction of surface hydrophilicity, metal conductivity and two-dimensional layered atomic structure [59], MXenes can be used to sense methanol and other gases, glucose, dopamine and nitrite in the field of biosensors [60].

Recently, several different compositions of MXenes have been proved to be biocompatible, with no cytotoxicity detected in living nerve tissue [61, 62]. Two-dimensional Ti_3_C_2_ MXene is also used in nerve recording in vivo, providing a high-resolution neural interface for neuroelectronic devices. Dissociated cortical neurons cultured on Ti_3_C_2_ could adhere normally, grow neurites and form neural networks [31]. In addition, NSCs cultured on laminin-coated Ti_3_C_2_T_x_ MXene nanosheets formed stable adhesion, retained the ability of proliferation and showed neurite growth, increased differentiation rate and synaptic formation [63]. Therefore, from the existing research results, it can be clearly seen that the outstanding electrical and surface functional properties of MXenes make them have potentially important applications in nerve tissue engineering.

However, it remains unclear how MXenes interact with ion channels in close contact, and how the electrophysiological properties of neurons are impacted by it. In the present study, we prepared biocompatible and conductive Ti_3_C_2_T_x_ MXene films and cultured neural stem cells on them. We focused on dissecting the effects of MXenes on the ion channels of NSC-derived neurons and synaptic connections between them, expanding our knowledge on the electrophysiological mechanisms of MXenes in promoting the maturation of neurons and neural circuits.

## Materials and methods

### Ti_3_C_2_T_x_ MXene solution preparation

Multilayer Ti_3_C_2_T_x_ were synthesized by selective etching of the Al atomic layer in Ti_3_AlC_2_ in a mixture of HCl and LiF. Ti_3_AlC_2_ (1 g, MAX phase) was slowly introduced into the mixture of HCl (8 M, 10 mL) and LiF (1 g) in ice bath and then incubated at 40 °C for 30 h with stirring. With the Al layer removed, Ti_3_C_2_T_x_ solution was washed with deionized water and centrifuged at 4000 rpm for 10 min. The washing and centrifugation steps were repeated about 10 times until the pH reached ~ 6. The Ti_3_C_2_T_x_ was next mixed with deoxidized and deionized water and sonicated for 1 h in ice bath. After centrifugation at 4000 rpm for 20 min, the Ti_3_C_2_T_x_ MXene colloidal solution in water was collected as the dark supernatant, and then spin-coated on the tissue culture polystyrene (TCPS). The area of each TCPS (10 mm) is about 0.785 cm^2^, and the amount of Ti_3_C_2_T_x_ MXene is 6 μg.

### Ti_3_C_2_T_x_ MXene film characterization

Transmission electron microscopy images and corresponding SAED of Ti_3_C_2_T_x_ MXene flakes were obtained by transmission electron microscopy (TEM, JEOL JEM-2100F). The adhesion and growth of NSCs on Ti_3_C_2_T_x_ MXene were observed by scanning electron microscopy (SEM, Hitachi Regulus8100). Raman measurements of Ti_3_C_2_T_x_ MXene film were conducted on a confocal Raman microscope (Horiba LabRAM HR800) at room temperature with an excitation wavelength of 633 nm. X-ray photoelectron spectroscopy (XPS) was performed with a PHI QUANTERA II (760Zi-A 6 GHz) and the wide scanning of samples was selected in the range of 0–1200 eV. The contact angle measurements were taken on an OCA 15 plus (Dataphysics).

### NSCs extraction and culture

NSCs were taken from the left and right hippocampus of 18 days old mouse embryos, at a time when these cells are highly proliferate, followed the guidelines and agreements approved by the Animal Care and Use Committee of Southeast University. Briefly, blood vessels and meninges on the hippocampus were removed in phosphate-buffered saline (PBS) at 4 °C. Then PBS was fully removed and Accutase solution (Thermo, USA) was added to digest the hippocampus in a 5% CO_2_ incubator at 37 °C for 15 min. After Accutase solution was fully washed, DMEM-F12 medium containing 2% B27, 1% Penicillin–Streptomycin, 10 ng/mL EGF (Thermo, USA) and 10 ng/mL FGF (Pepro tech, USA) was added. Then, gently grinding was applied to the dissociated tissues with a pipette tip till they were dispersed as much as possible, and cells were filtered and transferred to culture flask (Corning, USA) for proliferation in the incubator at 37 °C. After the purification of the neurospheres for three generations, individual NSCs were seeded on either TCPS or monolayer Ti_3_C_2_T_x_ MXene (MXenes, TCPS spin-coated with Ti_3_C_2_T_x_ MXene) coated with laminin (Sigma, USA) at a density of 2 $\times$ 10^6^/mL, then the differentiation medium (Stemcell, Canada) was changed after 24 h of proliferation. The differentiation process lasted for 14 days, and the medium was changed every 3 days.

### Immunofluorescence

For viability test, cells were washed with PBS after three days in vitro in proliferation medium, transferred to DMEM/F12, containing 0.05% Calcein-AM and 0.025% EthD-1, in the incubator for 20 min, and then washed with DMEM/F12. After sealing with DAKO, cells were mounted for confocal imaging. For immunofluorescence staining, cells were washed with PBS at 7 DIV (differentiation in vitro), fixed in 4% paraformaldehyde for 50 min, blocked in PBS containing 2% BSA, and permeated with 0.1% TritonX-100 for 90 min. The cells were incubated with primary antibodies overnight at 4 °C, and then stained with DAPI (Solarbio, China) following incubation with secondary antibodies for 60 min. The antibodies used in these experiments include primary antibodies against β-tubulin (Beyotime, China), Nestin (Beyotime, China), MAP2 (Abcam, UK), Synapsin-1 (Cell Signaling Tech, USA) and PSD95 (Millipore, Germany). After sealing, cells were mounted for imaging under a Zeiss 700 laser scanning confocal microscope.

### Electrophysiological recordings

Cells grown on TCPS or MXenes were immersed in an oxygenated extracellular solution containing (in mM) 135 NaCl, 4 KCl, 2 CaCl_2_, 1 MgCl_2_, 10 HEPES, 10 D-glucose (pH 7.40, osmolarity 300 mOsm). The cells were visualized through a 60X water-immersion objective in an up-right microscope (Olympus), and patch-clamp recordings were made through an EPC10/2 amplifier (HEKA Electronics, Lambrecht Pfalz, Germany), driven by a PC computer running Patchmaster (HEKA Electronics). Recording pipettes were pulled from borosilicate glass capillaries (Sutter) and filled with an internal solution containing (in mM) 135 K-methane sulfonate, 20 KCl, 2 EGTA, 3 Mg-ATP, 10 HEPES, and 0.5 Na-GTP (pH 7.20, osmolarity 300 mOsm). Cells were all held at − 90 mV and the uncompensated series resistance had a typical value of 6 ~ 10 MΩ. For experiments on voltage-gated Ca^2+^ or Na^+^ current, a Cs^+^-based internal solution containing (in mM) 135 Cs-methane sulfonate, 10 CsCl, 10 TEA-Cl, 10 HEPES, 2 EGTA, 3 Mg-ATP and 0.5 Na-GTP (pH 7.20, osmolarity 300 mOsm) was used. To examine the spiking behavior of these neurons, current steps of 500 ms with an amplitude of 10 ~ 100 pA were applied under the current-clamp mode. Between stimulations, a negative leaking current was injected to maintain a membrane potential of ~ − 90 mV, and spontaneous action potentials and resting membrane potentials were accessed by holding cells at zero current. Signals were filtered at 2 kHz and sampled at 100 kHz. All patch-clamp experiments were performed at room temperature and the liquid junction potential (~ 10 mV) was corrected offline.

### Statistical analysis

Morphological analysis was carried out in Image J Pro Plus, and statistical analyses were performed in GraphPad Prism 7. A home-made macro written in Igor Pro 6.22 was used for detection of spontaneous synaptic events. Origin 2018 was used to draw activation and inactivation curves. Throughout the study, data are presented as mean ± SEM. Statistical significance was assessed by either two-way ANOVA test followed by Bonferroni multiple comparison test, or Student’s *t*-test, and p < 0.05 was considered significant.

## Results and discussion

### Ti_3_C_2_T_x_ MXene fabrication

**Fig. 1:**
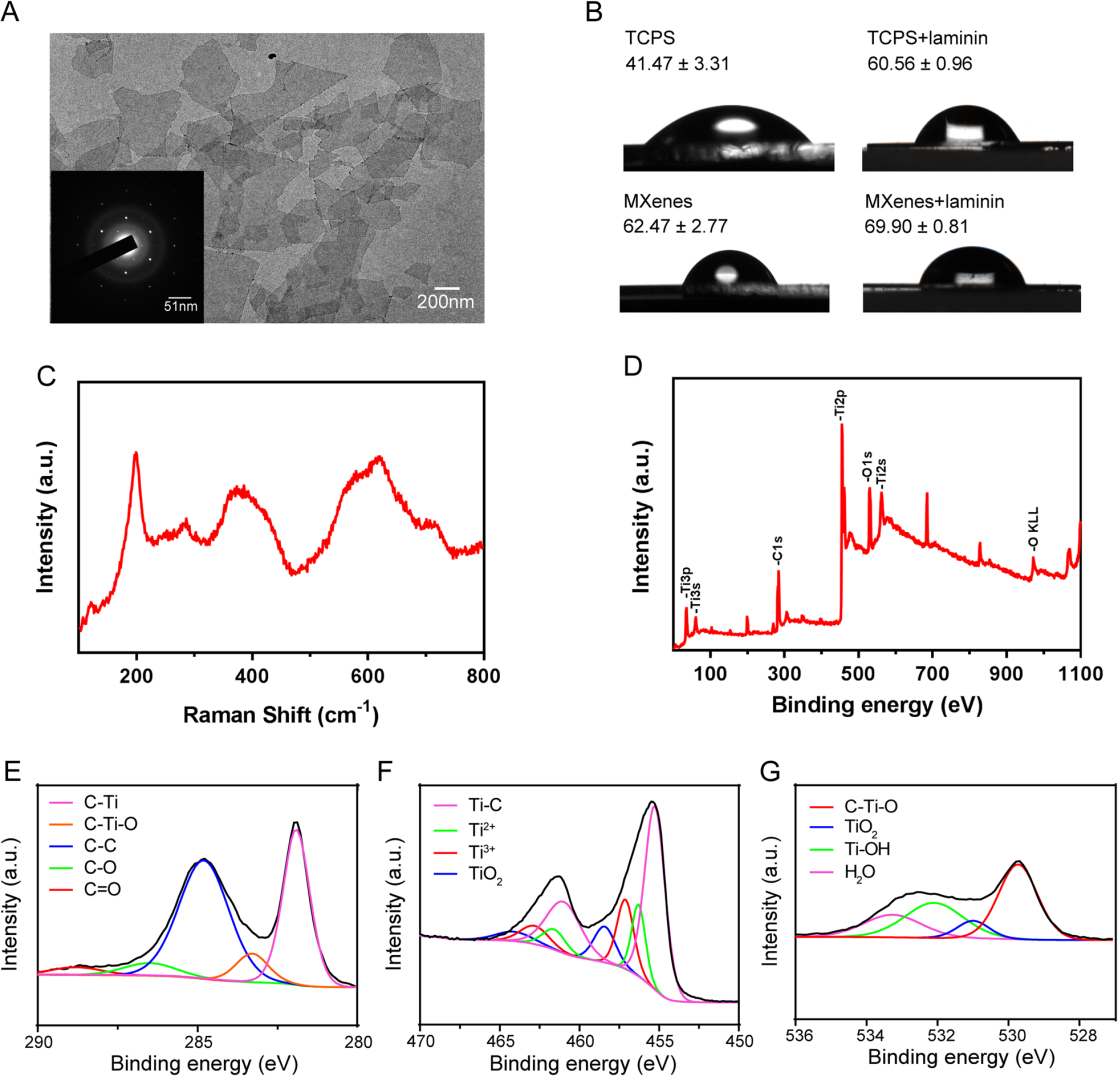
Characterization of Ti_3_C_2_T_x_ MXene. **A** Transmission electron microscopy (TEM) image of monolayer Ti_3_C_2_T_x_ MXene flakes. The inset shows a representative example of selected area electron diffraction (SAED). **B** Images of water contact angle on TCPS and Ti_3_C_2_T_x_ MXene before and after laminin treatment. **C** Raman spectroscopy of Ti_3_C_2_T_x_ MXene. **D** X-ray photoelectron spectroscopy (XPS) of Ti_3_C_2_T_x_ MXene. **E**–**G** XPS spectra of the C 1 s (**E**), Ti 2p (**F**), and O 1 s (**G**) regions

We fabricated Ti_3_C_2_T_x_ MXene through selective HF etching of the Al layer in Ti_3_AlC_2_ and characterized its morphology with transmission electron microscopy (TEM). As shown in the TEM image in Fig. 1A, the prepared MXene nanosheets display monolayer structure and two-dimension features with lateral sizes in the range of 100 nm ~ 800 nm, and the inset on the TEM image depicts a representative example of selected area electron diffraction (SAED), demonstrating a hexagonal crystal structure of Ti_3_C_2_T_x_ MXene. The structure and chemical composition of Ti_3_C_2_T_x_ MXene was investigated by Raman spectroscopy and X-ray photoelectron spectroscopy (XPS). Figure 1B shows the contact angle of water droplets on the nanosheets (62.47° ± 2.77°) is steeper when compared to that on the surface of tissue culture polystyrene (TCPS, 41.47° ± 3.31°), which may be due to the large surface roughness of Ti_3_C_2_T_x_ MXene. After coating with laminin, there was almost no difference in the hydrophilicity of MXenes surface (69.90° ± 0.81°), and was as close as possible to the coated TCPS (60.56° ± 0.96°). As shown in Fig. 1C, Raman fingerprint of Ti_3_C_2_T_x_ MXene was located in the range of 100 ~ 800 cm^−1^. The peaks at 199 and 715 cm^−1^ were the A_1g_ symmetry out-of-plane variation of Ti and C atoms, respectively. Furthermore, the peaks at 286, 369 and 623 cm^−1^ were the E_g_ group vibration of Ti, C and surface functional group atoms, respectively. In addition, the image of the nanosheets taken with X-ray photoelectron spectroscopy (XPS) indicates mainly diffraction peaks of such elements, including Ti 2 s, Ti 2p, O 1 s, C 1 s, and F 1 s, as expected from Ti_3_C_2_T_x_ MXene (Fig. 1D). Figure 1E–G displayed the C1s, Ti 2p, and O 1 s XPS spectra of Ti_3_C_2_T_x_ MXene as well. Finally, for hydrophilic test, the contact angles of water droplets on different surface states were measured after laminin coating. Taken together, these results demonstrated successful fabrication of Ti_3_C_2_T_x_ MXene in our hands. All of the following experimental TCPS and MXenes have been treated with laminin.

### Ti_3_C_2_T_x_ MXene promotes differentiation and morphological maturation of NSCs

It has been reported previously NSCs can adhere, grow and differentiate into neurons to form neural networks on MXenes and survive up to at least 7 days [63]. However, it is not clear if NSCs on MXenes can continue to develop to functionally mature stages. Therefore, we isolated NSCs from mouse hippocampus and stained for nestin, and the immunofluorescence images showed that almost all cells were nestin positive, indicating that the extracted cells were NSCs (Additional file 1: Fig. S1). The NSCs were then seeded on TCPS and Ti_3_C_2_T_x_ MXene substrate, respectively. The Live/Dead assay demonstrated that the Ti_3_C_2_T_x_ MXene displayed great cytocompatibility (Additional file 2: Fig. S2A and 2B). Afterwards, SEM was used to observe the adhesion, axon elongation and growth on TCPS and Ti_3_C_2_T_x_ MXene (Additional file 2: Fig. S2C). It was suggested that the NSCs grown on Ti_3_C_2_T_x_ MXene had similar growth morphology to those on TCPS. Subsequently, we conducted differentiation assay to examine the morphological and electrophysiological properties of neurons derived from NSCs.

**Fig. 2:**
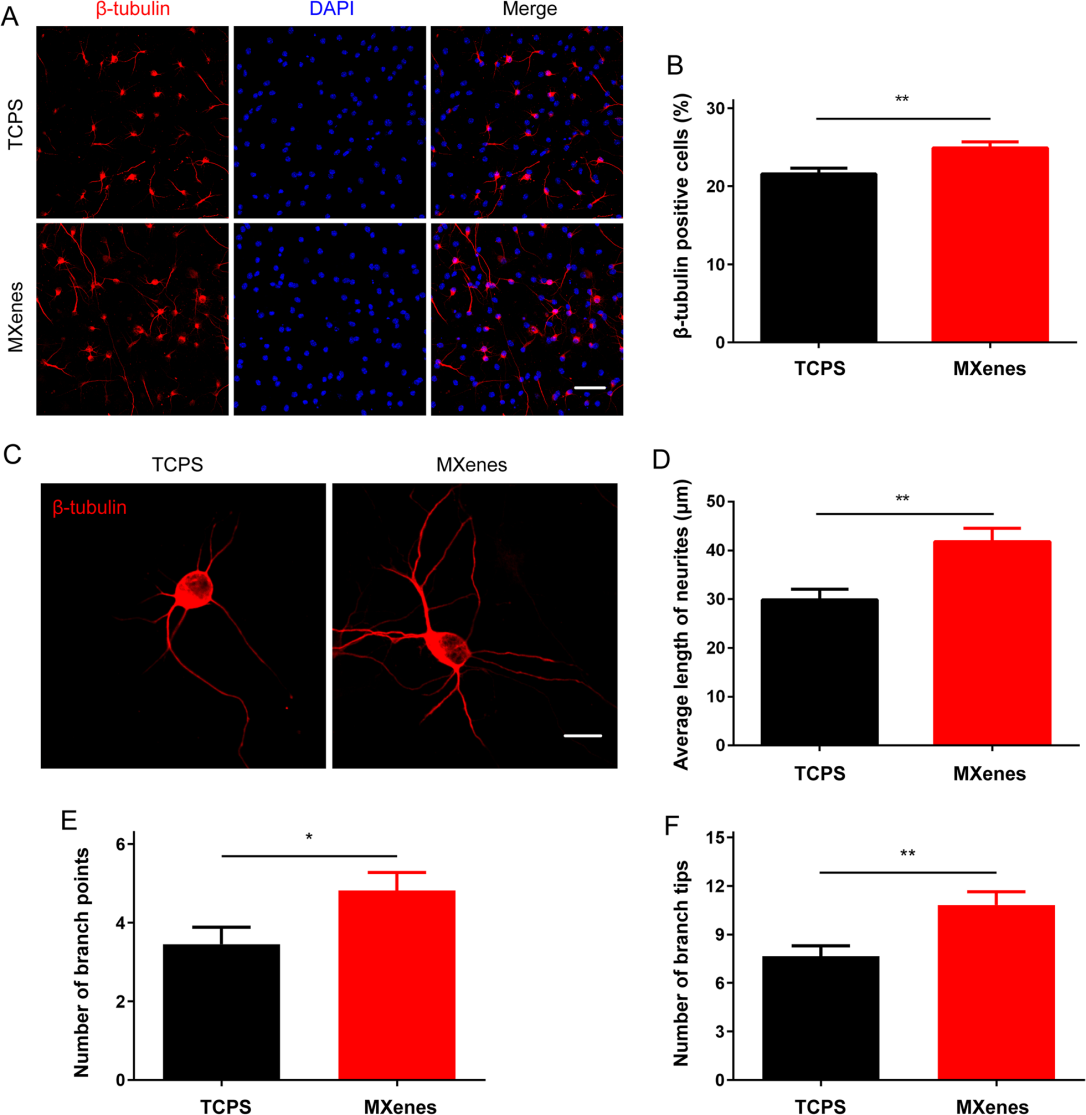
Ti_3_C_2_T_x_ MXene promoted NSCs development and growth. **A** and **B** Representative images of NSC-derived neurons stained with β-tubulin antibody and DAPI at 7 DIV. Scale bar = 50 μm. (**A**), and the percentage of β-tubulin positive cells on TCPS and MXenes (**B**). **C**–**F** Two representative new generated neurons cultured on TCPS and MXenes. Scale bar = 10 μm. (**C**), the average length of neurites (**D**), the number of branch points (**E**), and the number of branch tips (**F**) for new neurons cultured on TCPS and MXenes. * indicates p < 0.05, ** indicates p < 0.01

After 7 days of differentiation, the cells were stained with β-tubulin, a specific marker for neurons (Fig. 2A). Compared to those seeded on TCPS, the proportion of NSC-differentiated neurons (i.e. β-tubulin positive cells) on Ti_3_C_2_T_x_ MXene was increased significantly (TCPS: 21.57 ± 0.73%, n = 16 cultures; MXene: 24.90 ± 0.76%, n = 18 cultures; p < 0.01, Fig. 2B), and the MAP2 positive cells on Ti_3_C_2_T_x_ MXene was increased as well (TCPS: 25.18 ± 0.92%, n = 10 cultures; MXene: 30.12 ± 0.92%, n = 12 cultures; p < 0.01, Additional file 3: Fig. S3). In addition, we found that neurons grown on MXenes exhibited typical morphological maturation with neurite extension (Fig. 2C). Remarkably, the average length of neurites on MXenes was significantly longer (TCPS: 29.84 ± 2.19 μm, n = 11 cells; MXenes: 41.75 ± 2.75 μm, n = 13 cells; p < 0.01, Fig. 2D), and the numbers of branch points (TCPS: 3.45 ± 0.43, n = 11 cells; MXenes: 4.82 ± 0.46, n = 11 cells; p < 0.05, Fig. 2E) and branch tips (TCPS: 7.64 ± 0.66, n = 11 cells; MXenes: 10.82 ± 0.82, n = 11 cells; p < 0.01, Fig. 2F) on MXenes increased significantly as well. Consistent with a previous study [63], our results demonstrated that MXenes could significantly promote the differentiation of NSCs into neurons and even increase the length of their neurites.

### Ti_3_C_2_T_x_ MXene increases voltage-gated Ca^2+^ current in NSC-derived neurons

During the development and maturation of neurons, they must acquire a variety of ion channels that are essential for spiking and synaptic transmission [17, 64–66]. Given the close contact of Ti_3_C_2_T_x_ MXene with the plasma membrane of neurons cultured on it, it is conceivable that ion channels imbedded in the plasma membrane could be altered by Ti_3_C_2_T_x_ MXene, directly or indirectly. To address this question, we decided to perform patch-clamp recording on NSC-derived neurons cultured on TCPS or Ti_3_C_2_T_x_ MXene and investigate the currents flew through voltage-gated Na^+^, K^+^ and Ca^2+^ ion channels.

**Fig. 3:**
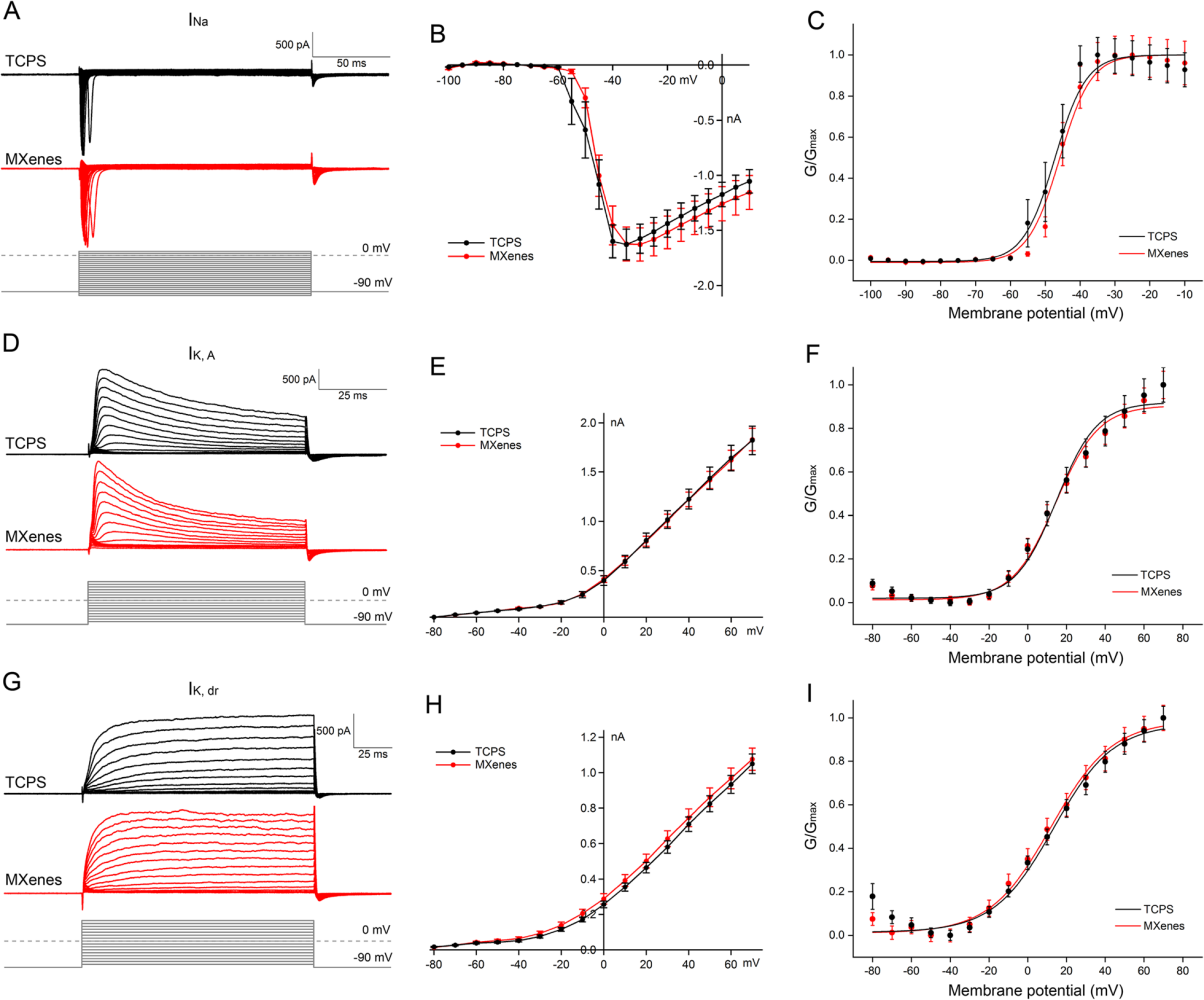
Ti_3_C_2_T_x_ MXene bore no effect on the activation of I_Na_ or I_K_. **A**–**C** Representative I_Na_ recorded in two NSC-derived neurons under voltage-clamp, in response to voltage steps (from − 100 mV to + 10 mV, 150 ms, 5 mV a step). Their current–voltage (IV) and activation curves, pooled from multiple cells, are shown in B and C, respectively. **D**–**F** I_K,A_ recorded under voltage-clamp, induced through voltage steps (− 80 ~ + 70 mV, 80 ms, 10 mV a step). **G**–**I** I_K,dr_ recorded under voltage-clamp, in response to the same voltage steps as in **D**. Neither the amplitude (**B**, **E** and **H**) nor the activation (**C**, **F** and **I**) of I_Na_, I_K,A_ or I_K,dr_ was significantly altered by Ti_3_C_2_T_x_ MXene

First, we examined voltage-gated Na^+^ current (I_Na_), which is essential for the depolarization phase of spikes [17]. To isolate I_Na_ from the whole-cell current, we used a Cs^+^-based internal solution and included 2 mM 4-AP and 10 mM TEA-Cl in the external solution to block voltage-gated K^+^ current (I_K_), and added 0.2 mM CdCl_2_ in the external solution to block voltage-gated Ca^2+^ current (I_Ca_). After breaking into the whole-cell mode, we held the cell at − 90 mV under voltage-clamp, applied voltage steps of − 100 ~ + 10 mV, 5 mV a step, for 150 ms, and recorded the resulting currents (Fig. 3A). As shown in the current–voltage (IV) curve plotted in Fig. 3B, I_Na_ recorded from neurons on both TCPS and Ti_3_C_2_T_x_ MXene was activated rapidly at about − 60 mV and reached the peak at − 40 ~ − 30 mV, and there is no significant difference in the peak amplitude between the two groups (TCPS: 1.63 ± 0.14 nA, n = 15; MXenes: 1.63 ± 0.15 nA, n = 18; p > 0.05).

**Fig. 4:**
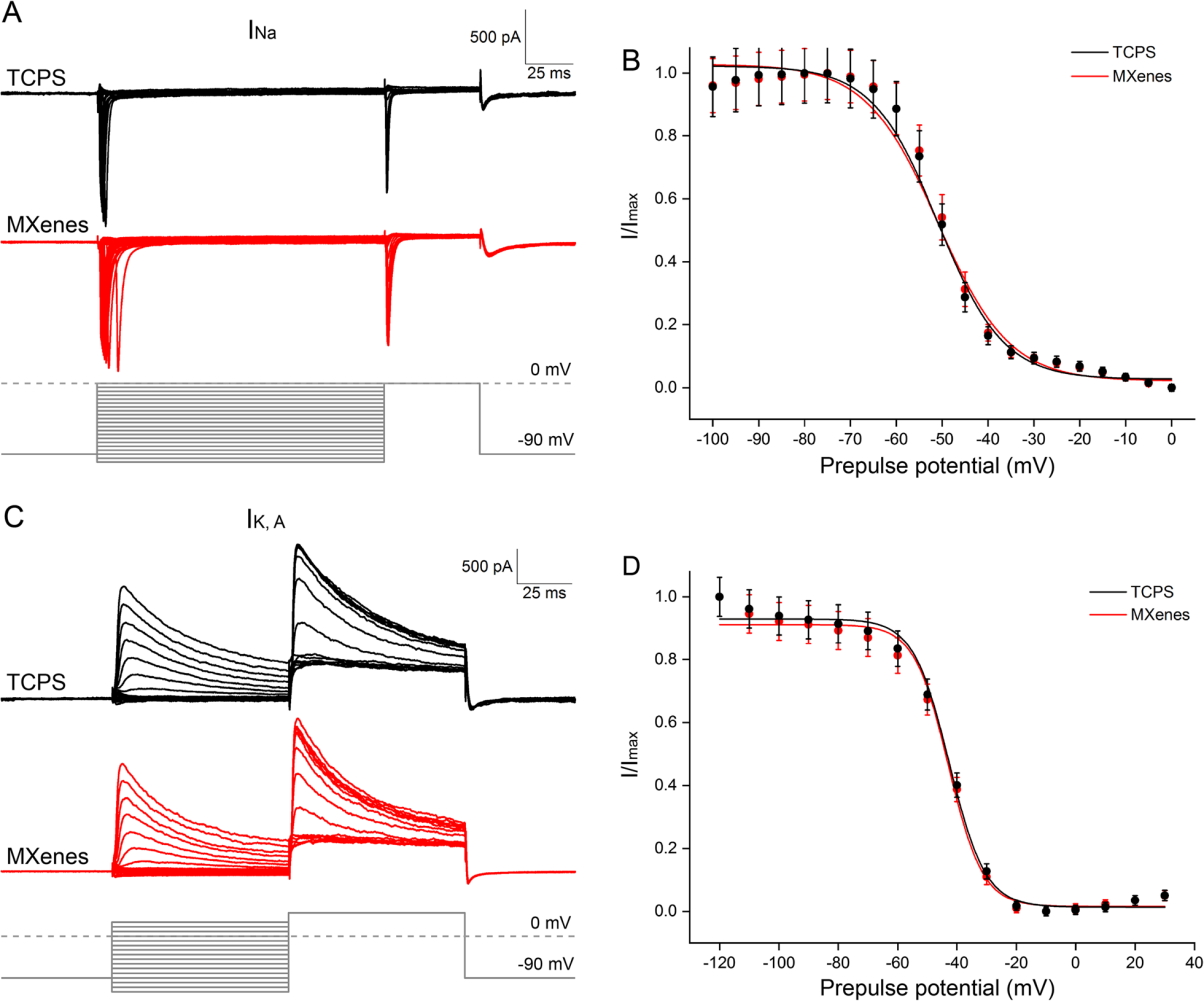
Ti_3_C_2_T_x_ MXene had no effect on the inactivation of I_Na_ or I_K,A_. **A** and **B** I_Na_ recorded in two NSC-derived neurons under voltage-clamp, in response to double-pulse stimulation (conditioning pre-pulse: from − 100 mV to 0 mV, 150 ms, 5 mV a step; depolarizing test pulse: 0 mV for 50 ms). **C** and **D** I_A_ recorded under voltage-clamp, in response to double-pulse stimulation (pre-pulse: from − 120 mV to + 30 mV, 80 ms, 10 mV a step; test pulse: + 50 mV for 80 ms)

We then converted current into conductance and fitted the activation of I_Na_ with a Boltzmann equation:

$${\text{G}}/{\text{G}}_{\text{max}}\text{=1/}\left\{{1} \, \text{+} \, {\text{exp}}\left[\left({\text{V}}_{\text{m}}-{\text{V}}_{1/2}\right)\text{/k}\right]\right\}$$

where G is the conductance, V_m_ is the membrane potential, V_1/2_ is the half-activation potential, and k is the slope factor (shown in Fig. 3C). We found that there is no significant difference in V_1/2_ or k between TCPS and MXene groups (TCPS: V_1/2_ = − 47.85 ± 0.43 mV, k = 3.51 ± 0.37, n = 15; MXenes: V_1/2_ = − 45.67 ± 0.14 mV, k = 2.83 ± 0.13, n = 18; p > 0.05), suggesting the activation of voltage-gated Na^+^ channels were not significantly altered by Ti_3_C_2_T_x_ MXene. Next, we studied the inactivation of I_Na_ with double-pulse stimulation: 150 ms conditioning pre-pulse from − 100 to 0 mV in 5 mV increment, followed by a 50 ms depolarizing test pulse to 0 mV (as shown in Fig. 4A). The peak amplitudes of I_Na_ were normalized and the inactivation curve was fitted with the same equation above (Fig. 4B). We found that ${\text{V}}_{1/2}$ and k of I_Na_ inactivation in the TCPS group was comparable to that of the MXene group (TCPS: V_1/2_ = − 50.92 ± 1.46 mV, k = 7.12 ± 0.83, n = 23; MXenes: V_1/2_ = − 50.96 ± 1.92 mV, k = 7.82 ± 0.98, n = 25; p > 0.05), indicating the inactivation of voltage-gated Na^+^ channels was not significantly altered by Ti_3_C_2_T_x_ MXene either.

Second, we turned to voltage-gated K^+^ current (I_K_), which dictates the repolarization phase of spikes, and to a large extent, repetitive spiking, mainly control the shape of the action potential, adjust the frequency of the action potential and the resting membrane potential [17]. To isolated the total I_K_, we included 1 μM tetrodotoxin (TTX) and 0.2 mM CdCl_2_ in the external solution to block I_Na_ and voltage-gated Ca^2+^ current, respectively. In addition, a subtype specific I_K_ blocker, TEA-Cl or 4-AP was also included to isolated the transient K^+^ current (I_K,A,_ sensitive to 4-AP at 2 mM) and delayed rectifier K^+^ current (I_K,dr_, sensitive to TEA-Cl at 10 mM), respectively. As shown in Fig. 3, neither I_K,A_ nor I_K,dr_ was significantly altered by Ti_3_C_2_T_x_ MXene, in terms of the peak amplitude or activation. Given that I_K,dr_ exhibited only minimal inactivation, we examined the inactivation of I_K,A_ only, and we once again found that it was not significantly altered (Fig. 4). Both I_Na_ and I_K_ are critically important for spiking, a central functional requirement for neurons. The fact that no significant alteration by Ti_3_C_2_T_x_ MXene was observed in either current, by far is the ultimate testament of Ti_3_C_2_T_x_ MXene’s safety and biocompatibility.

**Fig. 5:**
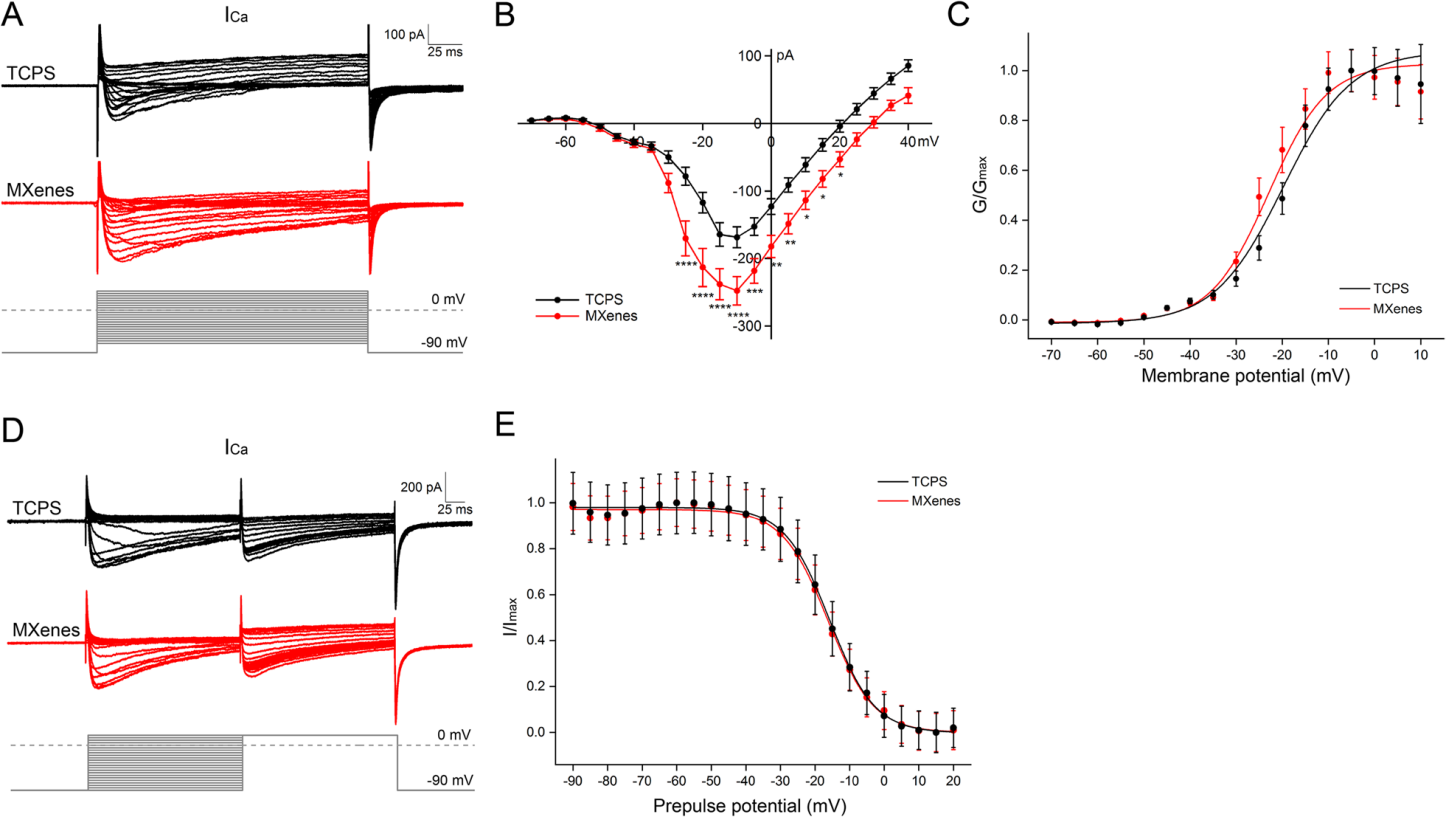
Ti_3_C_2_T_x_ MXene increased the amplitude of I_Ca_ without changing its voltage-dependent activation or inactivation. **A**–**C** Representative I_Ca_ recorded in two NSC-derived neurons under voltage-clamp, in response to voltage steps from -70 mV to + 40 mV, 200 ms, 5 mV a step, and their IV and activation curves, with data pooled from multiple cells. **D** and **E** I_Ca_ recorded under voltage-clamp, induced by double-pulse stimulation (pre-pulses: − 90 ~ + 20 mV, 200 ms, 5 mV a step; test pulse: + 20 mV for 200 ms). * indicates p < 0.05, ** indicates p < 0.01, *** indicates p < 0.001, **** indicates p < 0.0001

Lastly, we studied voltage-gated Ca^2+^ current (I_Ca_), which controls neurotransmitter release and regulates many other neuronal functions, including excitability and axon growth [17]. To isolated I_Ca_, we used a Cs^+^-based internal solution and added TTX (1 μM), TEA-Cl (10 mM) and 4-AP (2 mM) in the external solution to block I_Na_ and I_K_. When depolarization steps of − 70 ~ + 40 mV (5 mV a step) were applied to cells under voltage-clamp, small resulting inward currents were recorded (Fig. 5A), and the IV curve was constructed. As shown in Fig. 5B, I_Ca_ in NSC-derived neurons on both TCPS and MXenes was activated at about − 60 mV. However, I_Ca_ in neurons cultured on MXenes has a significantly larger amplitude at multiple voltages (For a voltage step of -10 mV, TCPS: 168.5 ± 15.5 pA, n = 45; MXenes: 248.1 ± 21.0 pA, n = 41; p < 0.0001). In alignment with our analysis on I_Na_ and I_K_, we also examined the activation and inactivation of I_Ca_, and we found neither of the two characteristics was significantly altered by Ti_3_C_2_T_x_ MXene (Fig. 5). Besides depolarizing the membrane potential, Ca^2+^ influx from I_Ca_ serves as triggers for a variety of intracellular signal transduction cascades, including synaptic vesicle release [67] and axon growth [68–70]. For establishing the relationship between Ca^2+^ influx and neurites growth, as previously reported [71–73], Ca^2+^ influx was increased by depolarizing with elevated extracellular K^+^ (10 mM KCl). After increasing intracellular Ca^2+^, the average length of neurites of NSC-derived neurons cultured on TCPS was significantly longer, (TCPS + 0 mM KCl: 55.15 ± 2.12 μm, n = 20 cells; TCPS + 10 mM KCl: 79.43 ± 4.15 μm, n = 23 cells; p < 0.0001, Additional file 4: Fig. S4) and comparable to the length of neurites cultured on MXenes without extra Ca^+^ influx (MXenes + 0 mM KCl: 88.43 ± 4.53 μm, n = 10 cells), suggesting MXenes lengthen neurites through increasing Ca^2+^ influx. Furthermore, there was no additional influence on neurites elongation on Ti_3_C_2_T_x_ MXene film after adding K^+^ to elevate intracellular Ca^2+^ (MXenes + 10 mM KCl: 91.98 ± 4.99 μm, n = 12 cells), indicating that the effects of MXenes on promoting neurite growth and maturation of neurons may be mediated merely through increasing Ca^2+^ influx. Our finding of an increased I_Ca_ on Ti_3_C_2_T_x_ MXene could contribute to the longer neurites of neurons described above, as well as the boosted spiking and enhanced synaptic transmission that are to be followed.

### Ti_3_C_2_T_x_ MXene boosts spiking of NSC-derived neurons

**Fig. 6:**
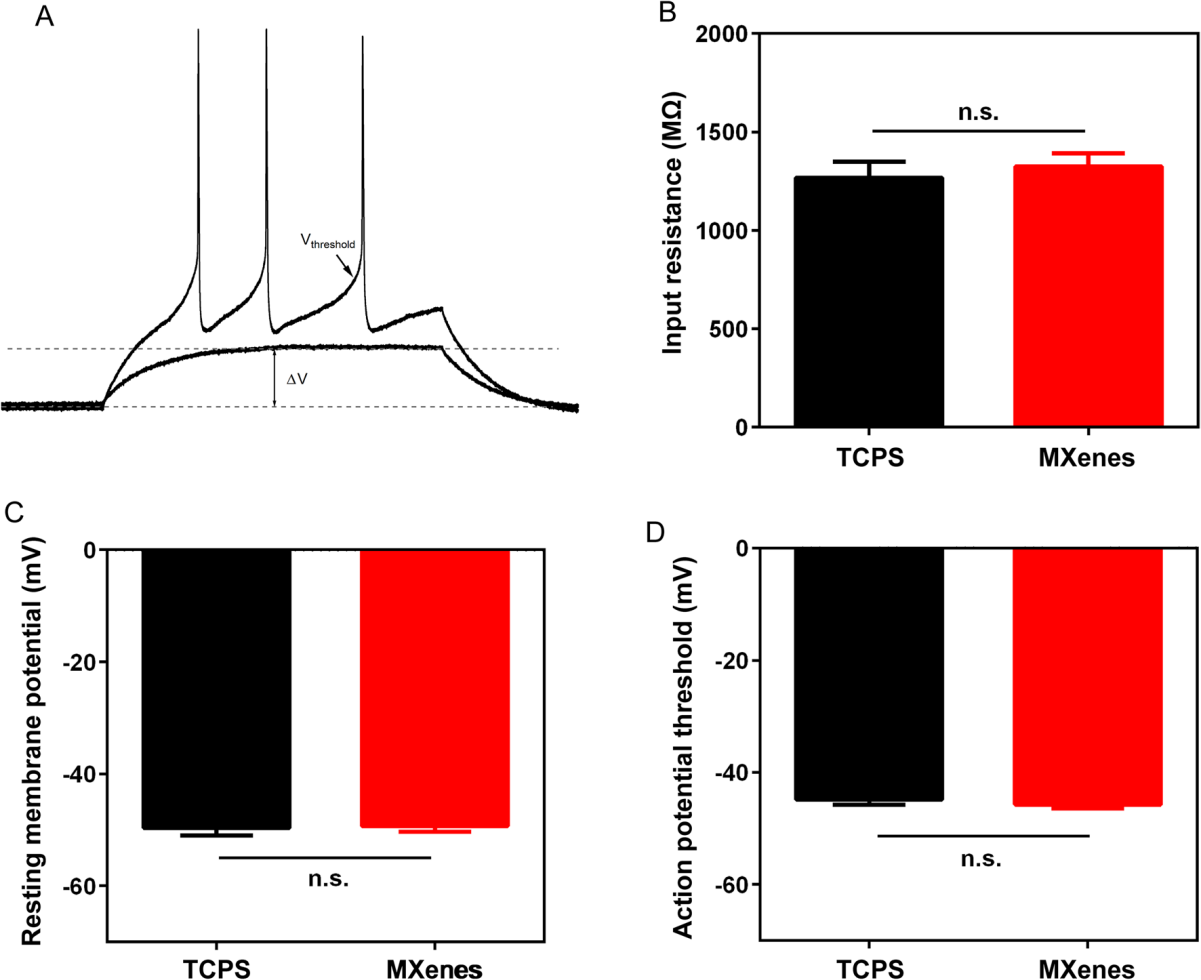
Passive membrane properties of NSC-derived neurons cultured on TCPS and MXenes after 7 DIV. **A** Representative voltage responses to step current injection, recorded from a single NSC-derived neuron. **B** Input resistance of new neurons cultured on TCPS and MXenes, determined by taking the largest voltage response with no spikes triggered and dividing the voltage change with the current injected (see the lower trace in **A**). **C** Resting membrane potential determined by holding cells at zero current. **D** Action potential threshold measured at the inflex point before an action potential (see the upper trace in **A**)

In addition to expression of voltage-gated currents, the therapeutic potential of NSCs relies on their continuation of development and maturation to acquire capabilities to fire action potential spikes and form synaptic connections. We therefore decided to examine the electrophysiological properties of NSC-derived neurons cultured on Ti_3_C_2_T_x_ MXene from 8 to 14 DIV. Each day, we picked one batch of NSC-derived neurons and performed whole-cell patch-clamp recording. We first focused on their passive membrane properties. To this end, we kept cells under current-clamp, injected step currents and recorded voltage responses (Fig. 6). Based on these voltage responses, we determined the input resistance, resting membrane potential and action potential threshold, which are widely considered to be indicators of cell maturation and health [74]. We found that there is no significant difference between neurons cultured on TCPS or Ti_3_C_2_T_x_ MXene in any of the three parameters: the input resistance (TCPS: 1267 ± 82.28 MΩ, n = 57 cells; MXenes: 1325 ± 66.39 MΩ, n = 110 cells), resting potential (TCPS: − 49.65 ± 1.37 mV, n = 57 cells; MXenes: − 49.33 ± 1.02 mV, n = 110 cells) and action potential threshold (TCPS: − 44.84 ± 0.85 mV, n = 57 cells; MXenes: − 45.70 ± 0.68 mV, n = 110 cells), consistent with previous studies [27, 75–77].

**Fig. 7:**
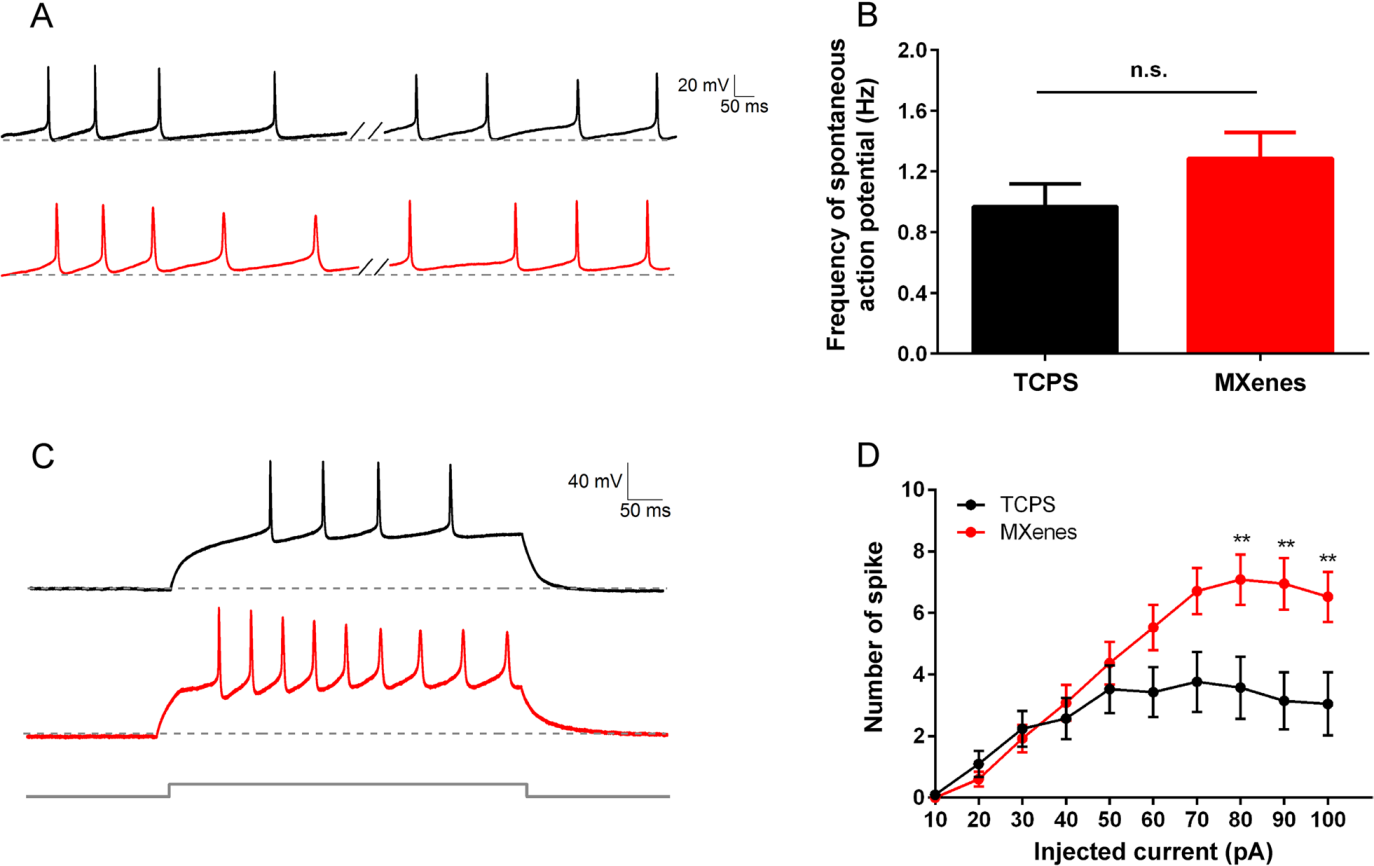
Ti_3_C_2_T_x_ MXene selectively enhanced NSC-derived neurons spiking under strong depolarization. **A** Representative spontaneous spikes recorded from two new generated neurons cultured on TCPS (black) and MXenes (red), by holding the cells at zero current. **B** Frequency of spontaneous spikes in new neurons cultured on TCPS and MXenes. **C** Representative voltage responses evoked by current injection, recorded from two new neurons cultured on TCPS (black) and MXenes (red). **D** Number of spikes evoked by step current of 10 ~ 100 pA. ** indicates p < 0.01

Next, we investigated the spiking pattern of neurons cultured on TCPS and Ti_3_C_2_T_x_ MXene. We first recorded spontaneous action potentials by holding cells at zero current under the current-clamp mode (Fig. 7A). We found that neurons cultured on Ti_3_C_2_T_x_ MXene fired spontaneous spikes as frequently as those on TCPS (TCPS: 0.97 ± 0.151 Hz, n = 62 cells; MXenes: 1.29 ± 0.17 Hz, n = 112 cells; Fig. 7B). We then applied step currents from 10 to 100 pA with 10 pA increment and recorded the spiking responses (Fig. 7C). We found that for step current injection of 10 ~ 70 pA, there is no significant difference in the spiking frequency between neurons cultured on TCPS or Ti_3_C_2_T_x_ MXene. This is consistent with the previous findings that no significant alteration by Ti_3_C_2_T_x_ MXene was observed in either I_Na_ and I_K_ (Figs. 3 and 4). For stronger current injection of 80 ~ 100 pA, however, neurons cultured on Ti_3_C_2_T_x_ MXene fired significantly more spikes when compared to those cultured on TCPS (For current injection of 80 pA, TCPS: 3.57 ± 1.01, n = 21 cells; MXenes: 7.08 ± 0.81, n = 38 cells; p < 0.01, Fig. 7D). While the exact underlying mechanisms are not clear, increased voltage-gated Ca^2+^ current in neurons cultured on Ti_3_C_2_T_x_ MXene, as described above, was likely to be involved in the boosted spiking observed in these neurons.

Taken together, our results on the passive membrane properties and spiking behavior strongly argue that Ti_3_C_2_T_x_ MXene are safe and highly compatible with living cells, making them an attractive biomaterial for constructing neural interface and scaffold. The increased spiking frequency for NSC-derived neurons cultured on Ti_3_C_2_T_x_ MXene in response to strong depolarization suggests Ti_3_C_2_T_x_ MXene is capable to facilitate or promote NSC development and maturation.

### Ti_3_C_2_T_x_ MXene enhances synaptic transmission between NSC-derived neurons

**Fig. 8:**
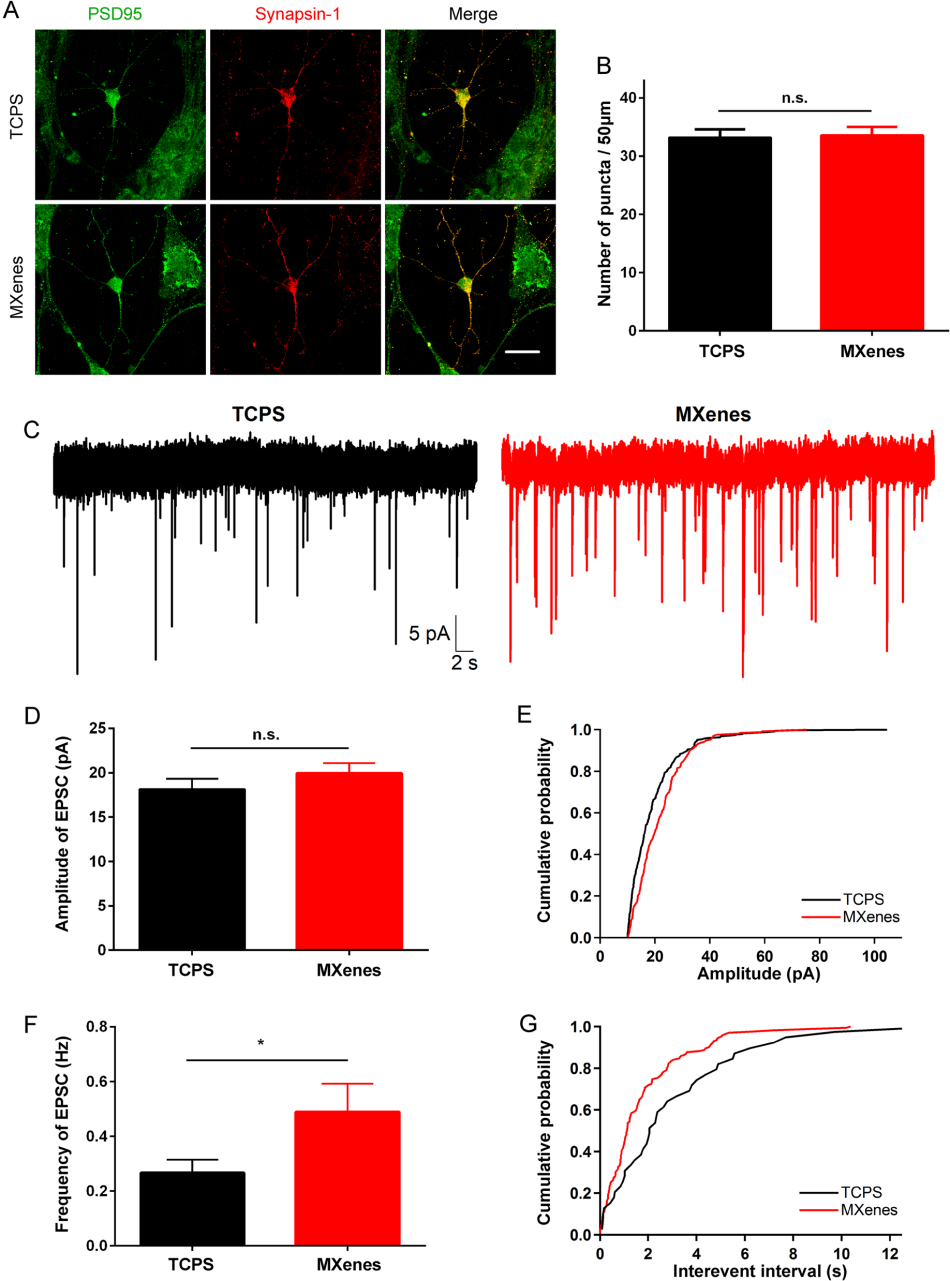
Ti_3_C_2_T_x_ MXene increased the frequency but not the amplitude of spontaneous synaptic currents in NSC-derived neurons. **A** and **B** Representative dual staining images of PSD95 and Synapsin-1 at 7 DIV for typical neuron cultured on TCPS and MXenes, Scale bar = 10 μm. (**A)** and the average number of synapses (**B**). **C** Typical spontaneous excitatory postsynaptic currents (EPSCs) recorded from two new neurons. **D** and **E** Average of EPSC amplitudes across different cells (**D**) and cumulative amplitude distribution of two new neurons (**E**). **F** and **G** Average of EPSC frequencies across different cells (**F**) and cumulative interevent interval distribution of two new neurons (**G**). * indicates p < 0.05

During the development of NSCs, synaptic connections are formed and strengthened [26]. We therefore decided to investigate if Ti_3_C_2_T_x_ MXene could bear any difference in these synaptic connections between NSC-derived neurons. First, we examined the number of synapses. Neurons derived from NSCs were co-stained with PSD95 and Synapsin-1, puncta of synapses can be found in these neurons grown on both TCPS and MXenes (Fig. 8A), and there is no significant difference in the number of synapses between the two groups (TCPS: 33.15 ± 1.43, n = 13 cells; MXenes: 33.57 ± 1.43, n = 14 cells; Fig. 8B). Next, we performed whole-cell patch-clamp recording and recorded spontaneous excitatory postsynaptic currents (EPSCs) from these neurons cultured on TCPS or MXenes. As shown in Fig. 8C, EPSCs were detected in both experimental groups, indicating that synaptic connections revealed in immunostaining are functional. Although there is no significant difference in the amplitude of ESPCs between the two groups (TCPS: 18.28 ± 1.28 pA, n = 48 cells; MXenes: 19.74 ± 1.18 pA, n = 41 cells; Fig. 8D and E), EPSCs recorded from neurons cultured on Ti_3_C_2_T_x_ MXene are more frequent (TCPS: 0.25 ± 0.05 Hz, n = 48 cells; MXenes: 0.50 ± 0.11 Hz, n = 41 cells; p < 0.05, Fig. 8F and G).

Taken together, these results show that regenerated neurons on Ti_3_C_2_T_x_ MXene could make functional synapses with one another and form mature neural networks, further reinforce its safety and high biocompatibility. Furthermore, Ti_3_C_2_T_x_ MXene enhances synaptic transmission by selectively increasing the frequency but not the amplitude of synaptic events, affording additional advantageous for Ti_3_C_2_T_x_ MXene to be used in neural interface and scaffold.

## Conclusion

In this study, we investigated the effects of two-dimensional Ti_3_C_2_T_x_ MXene film on the electrophysiological maturation of NSCs. Through the whole-cell patch-clamp recording technique, the direct effects of Ti_3_C_2_T_x_ MXene on NSC-derived neurons and its influence on the formation and performance of neural networks were examined. Ti_3_C_2_T_x_ MXene films with a thickness of several hundred nanometers were fabricated and spin-coated on TCPS, and they exhibited good biocompatibility as NSCs cultured on them are as viable as the control group. In addition, NSCs cultured on Ti_3_C_2_T_x_ MXene films differentiated into neurons with higher efficiency and longer neurites, highlighting their capability to promote NSCs maturation. Furthermore, close contact with Ti_3_C_2_T_x_ MXene bears no appreciative difference in voltage-gated Na^+^ or K^+^ current, but selectively increases the amplitude of voltage-gated Ca^2+^ current, which could contribute to the longer neurites observed. Finally, Ti_3_C_2_T_x_ MXene does not alter the passive membrane properties of matured neurons and promotes neuronal firing only for strong depolarization. Furthermore, Ti_3_C_2_T_x_ MXene does not change the number synapses or the amplitude of synaptic events, but it is capable to enhance synaptic transmission by increasing the frequency of synaptic releases. In summary, our results strongly suggest two-dimensional Ti_3_C_2_T_x_ MXene represents a new and promising direction for conductive neural interface or scaffold in stem cell therapy and nerve tissue engineering from morphology, physiology and functionality.

However, there are still limitations in our study. Firstly, we used laminin coating like other studies [7, 75, 78–81], which helps the adhesion of NSCs, but weakens the most direct influence of Ti_3_C_2_T_x_ MXene on NSCs. Secondly, in order to fully reveal the interaction between Ti_3_C_2_T_x_ MXene and neural tissue, it is necessary to accurately control its size, composition and surface functional groups and evaluate in a more systematic way its impact on functions of individual neurons and neural circuits among them. Finally, the physical interacting of MXenes with K^+^, Na^+^ and Ca^2+^ channels and the underlying mechanisms also need to be further studied [26].

## Supplementary Information

**Additional file 1: Figure S1.** Identification of NSCs. Representative images of NSCs sphere stained with Nestin (NSCs marker, green) and DAPI (nucleus, blue). Scale bar = 50 μm.

**Additional file 2: Figure S2**. Biocompatibility of Ti_3_C_2_T_x_ MXene for NSCs growth. (A) Representative images of NSCs stained with Calcein-AM and EthD-1 after 3 days in the proliferation phase. (B) Bar graph of NSCs viability on TCPS and MXenes (TCPS: 96.16 ± 0.64%, n = 6 cultures; MXene: 97.25 ± 0.56%, n = 6 cultures). Scale bar = 50 μm. (C) Representative SEM images of NSCs cultured on TCPS and Ti_3_C_2_T_x_ MXene film under low (scale bar = 100 μm) and high (scale bar = 100 nm) magnification.

**Additional file 3: Figure S3**. Maturation of NSC-derived neurons on Ti_3_C_2_T_x_ MXene. (A) Representative images of NSC-derived neurons stained with MAP2 and DAPI at 7 DIV. Scale bar = 50 μm. (B) The percentage of MAP2 positive cells on TCPS and MXenes. ** indicates p < 0.01.

**Additional file 4: Figure S4**. Effects of increased Ca^2+^ influx on the neurite length. (A) Representative images of NSC-derived neurons treated with 10 mM KCl on TCPS and MXene at 7 DIV. Scale bar = 50 μm. (B) The average length of neurites under different conditions. **** indicates p < 0.0001.

## Data Availability

The data that support the findings of this study are available from the corresponding author upon reasonable request.

## Abbreviations

NSCs: Neural stem cells
TCPS: Tissue culture polystyrene
SAED: Selected area electron diffraction
TEM: Transmission electron microscopy
XPS: X-ray photoelectron spectroscopy
PBS: Phosphate-buffered saline
EGF: Epidermal growth factor
FGF: Fibroblast growth factor
DIV: Differentiation in vitro
SEM: Scanning electron microscopy
MAP2: Microtubule-associated protein 2
