# Supplementary figures and images for: Two-dimensional Ti3C2Tx MXene promotes electrophysiological maturation of neural circuits
Source: J Nanobiotechnology. 2022 Aug 31;20:398. doi: 10.1186/s12951-022-01590-8 (PMC9434915; doi:10.1186/s12951-022-01590-8)

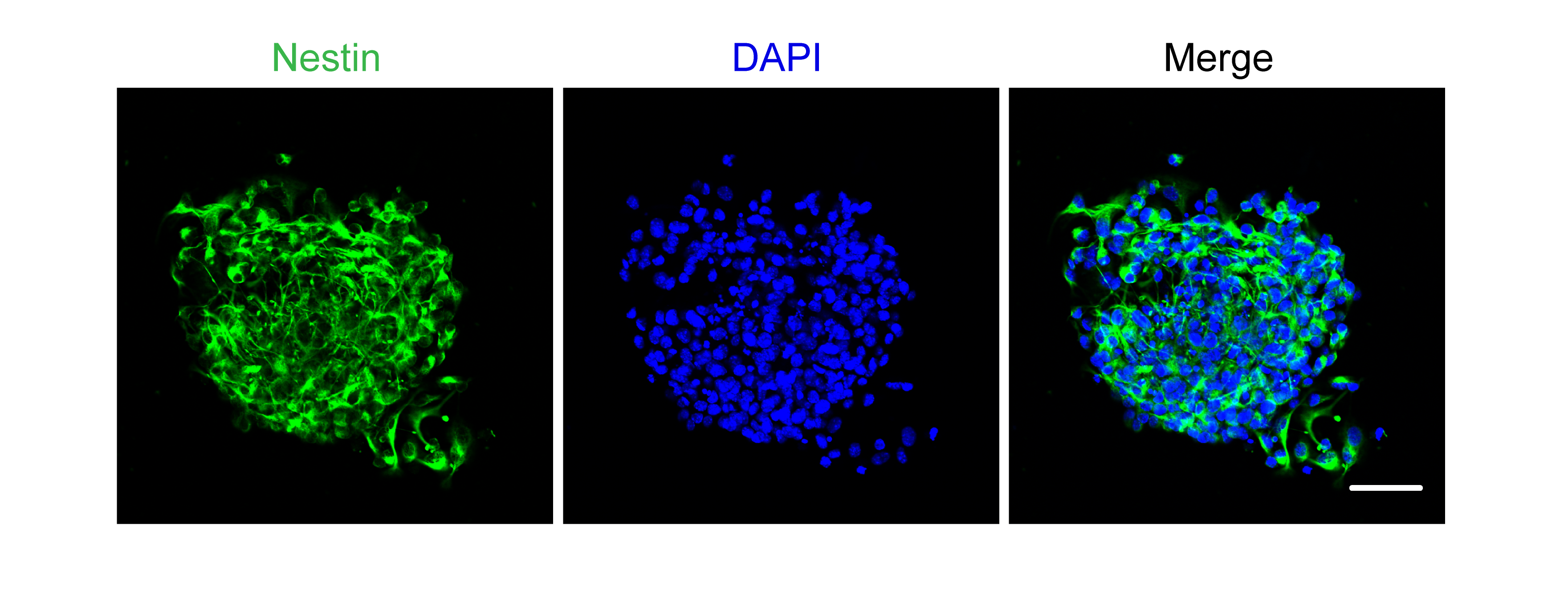

Supplement: Supplementary file 1 — Additional file 1: Figure S1. Identification of NSCs. Representative images of NSCs sphere stained with Nestin (NSCs marker, green) and DAPI (nucleus, blue). Scale bar = 50 μm. [file 12951_2022_1590_MOESM1_ESM.tif]

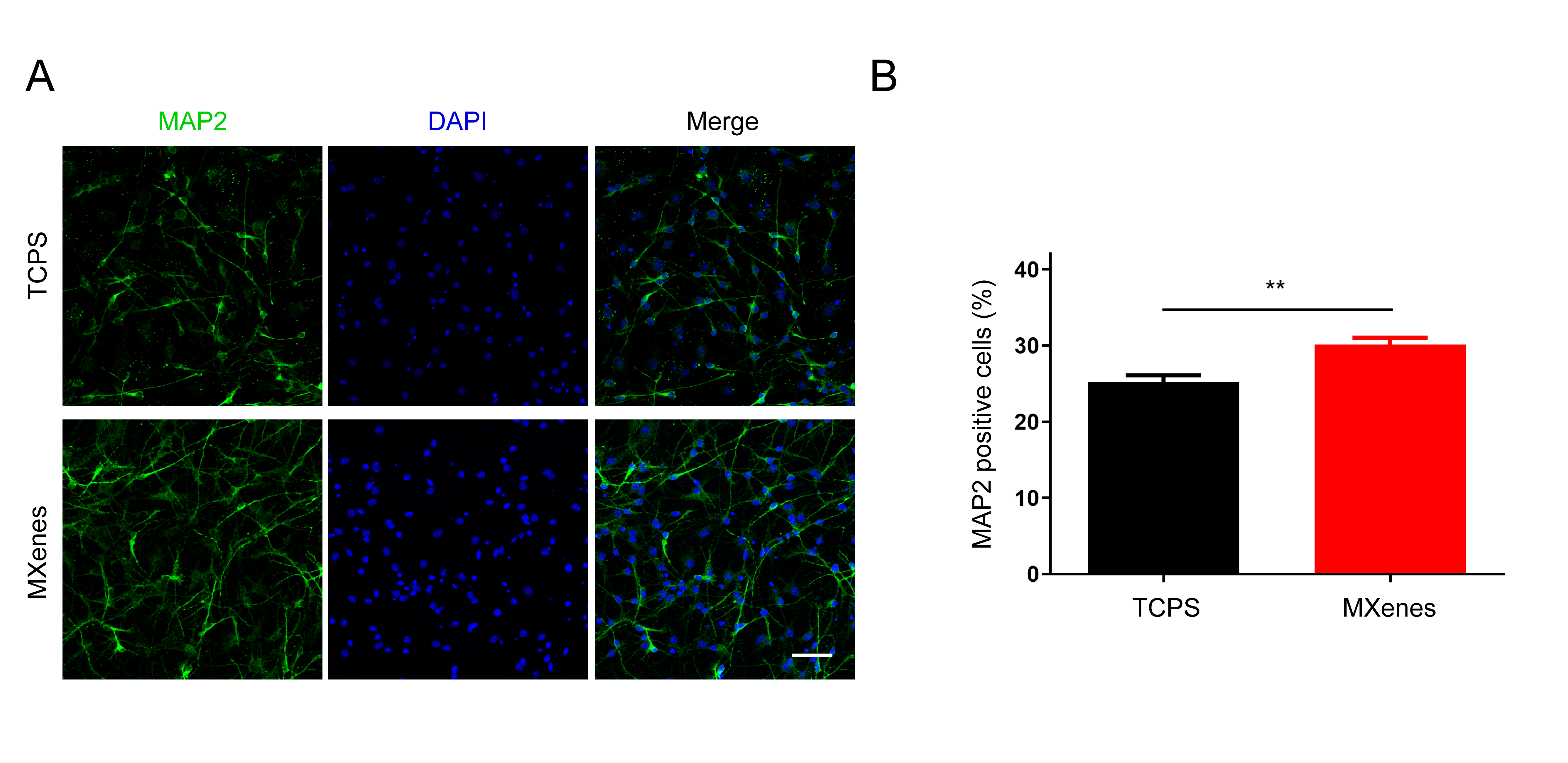

Supplement: Supplementary file 3 — Additional file 3: Figure S3. Maturation of NSC-derived neurons on Ti3C2Tx MXene. (A) Representative images of NSC-derived neurons stained with MAP2 and DAPI at 7 DIV. Scale bar = 50 μm. (B) The percentage of MAP2 positive cells on TCPS and MXenes. ** indicates p < 0.01. [file 12951_2022_1590_MOESM3_ESM.tif]

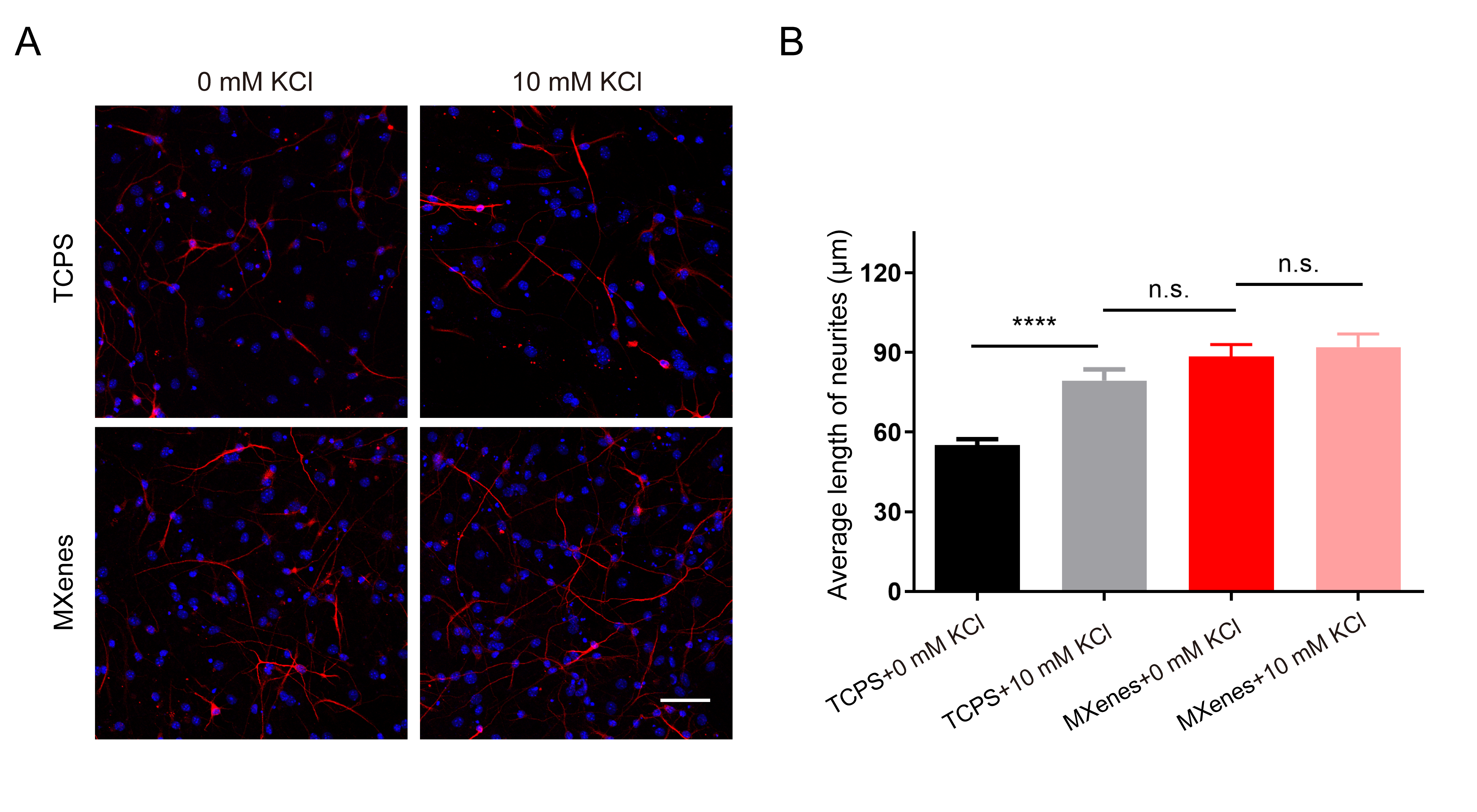

Supplement: Supplementary file 4 — Additional file 4: Figure S4. Effects of increased Ca2+ influx on the neurite length. (A) Representative images of NSC-derived neurons treated with 10 mM KCl on TCPS and MXene at 7 DIV. Scale bar = 50 μm. (B) The average length of neurites under different conditions. **** indicates p < 0.0001. [file 12951_2022_1590_MOESM4_ESM.tif]
